# Exendin-4 protects against post-myocardial infarction remodelling via specific actions on inflammation and the extracellular matrix

**DOI:** 10.1007/s00395-015-0476-7

**Published:** 2015-03-01

**Authors:** Emma Robinson, Roslyn S. Cassidy, Mitchel Tate, Youyou Zhao, Samuel Lockhart, Danielle Calderwood, Rachel Church, Mary K. McGahon, Derek P. Brazil, Barbara J. McDermott, Brian D. Green, David J. Grieve

**Affiliations:** 1 Centre for Experimental Medicine, Institute of Clinical Science Block A, Queen’s University Belfast, Grosvenor Road, Belfast, BT12 6BA UK; 2 School of Biological Sciences, Queen’s University Belfast, Belfast, BT9 5AG UK

**Keywords:** Glucagon-like peptide-1, Myocardial infarction, Cardiac remodelling, Extracellular matrix, Inflammation

## Abstract

Glucagon-like peptide-1 (GLP-1) is an insulin-releasing hormone clinically exploited for glycaemic control in diabetes, which also confers acute cardioprotection and benefits in experimental/clinical heart failure. We specifically investigated the role of the GLP-1 mimetic, exendin-4, in post-myocardial infarction (MI) remodelling, which is a key contributor to heart failure. Adult female normoglycaemic mice underwent coronary artery ligation/sham surgery prior to infusion with exendin-4/vehicle for 4 weeks. Metabolic parameters and infarct sizes were comparable between groups. Exendin-4 protected against cardiac dysfunction and chamber dilatation post-MI and improved survival. Furthermore, exendin-4 modestly decreased cardiomyocyte hypertrophy/apoptosis but markedly attenuated interstitial fibrosis and myocardial inflammation post-MI. This was associated with altered extracellular matrix (procollagen IαI/IIIαI, connective tissue growth factor, fibronectin, TGF-β_3_) and inflammatory (IL-10, IL-1β, IL-6) gene expression in exendin-4-treated mice, together with modulation of both Akt/GSK-3β and Smad2/3 signalling. Exendin-4 also altered macrophage response gene expression in the absence of direct actions on cardiac fibroblast differentiation, suggesting cardioprotective effects occurring secondary to modulation of inflammation. Our findings indicate that exendin-4 protects against post-MI remodelling via preferential actions on inflammation and the extracellular matrix independently of its established actions on glycaemic control, thereby suggesting that selective targeting of GLP-1 signalling may be required to realise its clear therapeutic potential for post-MI heart failure.

**Electronic supplementary material:**

The online version of this article (doi:10.1007/s00395-015-0476-7) contains supplementary material, which is available to authorized users.

## Introduction

Chronic heart failure (CHF) affects up to 2 % of the adult population in the western world and carries a poor prognosis, with the underlying aetiology shifting towards ischaemia [1, 2]. Acute remodelling in the infarct zone occurs within the first few days after myocardial infarction (MI) and involves apoptosis and inflammation which drive scar formation, whereas post-MI remodelling affecting the remote viable myocardium includes initial adaptive changes in cardiomyocyte biology and the extracellular matrix (ECM) progressing to maladaptive left ventricular (LV) dilatation, chronic myocardial inflammation, cardiomyocyte hypertrophy/apoptosis and interstitial fibrosis, which contribute to associated contractile dysfunction and ultimately CHF [3]. Despite optimal pharmacological and surgical management of post-MI patients there remains a substantial incidence of CHF [4, 5], so the need for more effective therapeutic strategies is clear.

In this regard, the incretin hormone, glucagon-like peptide-1 (GLP-1), is emerging as a potential candidate. Beyond its established role in glycaemic control, accumulating evidence supports other important physiological roles for GLP-1, particularly in the cardiovascular system [6, 7], where the GLP-1 receptor (GLP-1R) is widely expressed and mediates key functions including heart rate/contractility, cardiac structure/contractility, blood pressure and vascular tone [8–12]. Importantly, GLP-1 also confers beneficial actions on cardiovascular disease, both experimental and clinically, in the presence or absence of diabetes [13–18].

The majority of studies investigating cardiac effects of GLP-1 have focused on its actions in ischaemia and ability to protect cardiomyocytes from acute damage. For example, it is well established that GLP-1R agonists and dipeptidyl peptidase-4 (DPP-4) inhibitors, which increase endogenous GLP-1, reduce infarct size after experimental ischaemia and enhance functional recovery upon reperfusion [8, 19, 20]. Furthermore, the GLP-1R agonist, liraglutide, increases survival in experimental MI, and cardiac function after ischaemia–reperfusion injury is improved in DPP-4^−/−^ mice [15, 17]. GLP-1 also protects against LV dysfunction in experimental models of dilated cardiomyopathy, hypertensive CHF and MI, at least partly by increasing myocardial glucose uptake [14, 16, 21]. Importantly these benefits appear to translate to the clinical setting with GLP-1 improving cardiac function in CHF and in MI patients after primary angioplasty [18, 22].

Although the acute cardioprotective actions of GLP-1 are well established, little attention has previously been paid to specific effects on post-MI remodelling, which is a major contributor to CHF progression [3]. However, two studies have now reported beneficial actions of the GLP-1 analogues, AC3174 and exendin-4, on post-MI survival and cardiac structure/function in rodent models, when administered from 2 days and 2 weeks after coronary artery ligation, respectively [21, 23], although another study failed to demonstrate equivalent beneficial effects when liraglutide was given 7 days post-MI [24]. As previous work suggests that GLP-1 modulates several signalling pathways related to specific facets of post-MI remodelling, including cardiomyocyte apoptosis, fibrosis and inflammation [25–30], which are key determinants of CHF progression, a more detailed focus on individual remodelling components was considered to be crucial. The aim of this study was therefore to precisely define the apparently favourable actions of the peptide in this setting, testing the hypothesis that exendin-4 attenuates post-MI remodelling via differential modulation of distinct aspects of the remodelling phenotype. Importantly, we report for the first time specific beneficial actions of exendin-4 on post-MI remodelling, which appear to involve preferential targeting of the ECM secondary to modulation of myocardial inflammation.

## Methods

### Experimental model

Female C57BL/6J mice (8–12 weeks; Harlan UK) were used throughout this study and were housed under constant climatic conditions with free access to food and water. All experimental procedures were performed in accordance with the Guidance on the Operation of the Animals (Scientific Procedures) Act, 1986 (UK) and approved by the Queen’s University Belfast Animal Welfare and Ethical Review Body. Experimental animals were randomised (by drawing of lots) and subjected to permanent ligation of the left anterior descending coronary artery under 2 % isoflurane/oxygen anaesthesia, or sham surgery which involved an identical procedure with the exception of coronary artery ligation, and subsequent analyses performed at 4 weeks. Buprenorphine analgesia (0.05 mg/kg i.m.) was administered prior to and after surgery, as required. Female mice were used as previous studies have shown them to carry significantly lower peri-operative mortality versus males [31]. To investigate the effects of GLP-1 on post-MI remodelling, MI or sham-operated mice were randomly assigned to be chronically infused with either the DPP-4 resistant GLP-1 mimetic, exendin-4 (at a concentration employed in previous studies relating to the anti-diabetic and cardioprotective actions of GLP-1; 25 nmol/kg/day; GL Biochem, average purity 90 %) [12, 15, 16, 32], or saline control via an osmotic minipump (Alzet model 1004) implanted immediately after MI surgery. For ex vivo analysis of infarct size and gene/protein expression, animals were killed by sodium pentobarbitone overdose before hearts were excised and either perfused with Evan’s blue dye or non-infarcted tissue frozen in liquid nitrogen and stored at −80 °C for further studies. For analysis of post-MI remodelling, hearts were arrested in diastole by injection of 10 % KCl, fixed in 10 % neutral-buffered formalin solution, paraffin-embedded and sectioned. A minimum of five animals per group were studied for all protocols.

### Assessment of plasma glucose, insulin and lipids

Terminal blood samples were collected by cardiac puncture into heparinised tubes and centrifuged at 10,000*g* for 10 min to obtain plasma fractions. Plasma samples were analysed using enzymatic assay kits (Analox Ltd) for glucose (GMRD-002A using glucose oxidase), cholesterol (GMRD-084 using cholesterol esterase) and triglyceride (GMRD-195 using lipase) detected on a GM7 Micro-Stat Analyser (Analox Instruments Ltd). Plasma insulin concentrations were assayed using an ELISA kit (ALPCO Diagnostics), measuring absorbance at 450 nm on a microplate reader (Tecan Safire). Plasma glycated haemoglobin, HbA1c, was assessed using a commercially available assay kit (Helena Laboratories), measuring absorbance at 415 nm on a microplate reader (Tecan Safire) and expressed as percentage of total haemoglobin.

### Plasma adipokine expression

Adipokine protein expression was assessed in six pooled plasma samples from each experimental group using a Proteome Profiler™ antibody array (R&D Systems).

### Infarct size

Excised hearts were initially perfused retrogradely with Evans blue dye (1 % in saline) to determine area at risk. Hearts were then sliced into five serial transverse sections (1 mm) and incubated in 1 % triphenyltetrazolium chloride at 37 °C to identify infarcted myocardium. Infarct area, area at risk and total LV area from each section were measured using computerised planimetry (ImageJ), and totalled for all sections. Infarct size was expressed as a percentage of area at risk.

### Echocardiography and invasive assessment of cardiac function

Mice were anaesthetised with 1.5 % isoflurane/oxygen, placed on a warming pad, and imaged in the supine position using a Vevo770 ultrasound system with high-frequency 45 MHz RMV707B scanhead (VisualSonics, Inc.). M-mode parasternal short-axis scans at papillary muscle level were used to quantify LV end-diastolic (LVEDD) and end-systolic diameters (LVESD) from which  % fractional shortening was calculated (LVEDD − LVESD)/LVEDD × 100. Parasternal long-axis scans were used to provide additional data on LV end-diastolic (LVEDV) and end-systolic volumes (LVESV) and ejection fraction and pulse-wave Doppler was used to assess mitral valve flow (*E*/*A* ratio) as a measure of diastolic function. Isoflurane was then increased to 2 % before the right carotid artery was cannulated with a high-fidelity 1.2F pressure catheter (FTS-1211B-0018; Scisense Inc.), aortic pressure measured, and the catheter advanced into the LV for recording of steady-state function.

### Assessment of post-MI remodelling

Following killing, hearts were weighed and measurements normalised to total body weight. All histological analyses were performed on fixed (10 % neutral-buffered formalin), paraffin-embedded LV sections (5 μm). Cardiomyocyte cross-sectional area was determined by H&E staining, analysing cells with centrally located nuclei. Cardiac interstitial fibrosis was assessed by picrosirius red staining (0.1 % w/v), excluding coronary vessels and perivascular regions. Data were quantified by digital image analysis (NIS-Elements, Nikon) with the observer blinded to sample identity.

Immunohistochemistry for CD45 and F4/80 was performed with rabbit polyclonal (Millipore), rat polyclonal (BD Biosciences), and rat monoclonal (Abcam) antibodies (1:1000), respectively, using diaminobenzidine as the chromogen and nuclear counterstaining with haematoxylin. Data were quantified by blinded digital image analysis (NIS-Elements).

Cardiomyocyte apoptosis was determined by terminal deoxynucleotidyl transferase-mediated dUTP nick end labelling (TUNEL) staining (Roche Diagnostics). TUNEL-positive cardiomyocyte nuclei were counted, and data expressed as % total nuclei identified by 4′,6-diamidino-2-phenylindole staining in the same sections.

### In vitro cardiomyocyte remodelling studies

To investigate direct effects of exendin-4 on cardiomyocyte remodelling, a series of studies were conducted in rat ventricular H9c2 cardiomyoblasts and mouse atrial HL-1 cardiomyocytes, to assess actions on cell hypertrophy and apoptosis, respectively. H9c2 cardiomyoblasts were maintained in DMEM containing 10 % FCS, 100 U/ml penicillin and 100 µg/ml streptomycin. At passage, they were plated, cultured to ~50 % confluency and serum-starved for 24 h prior to incubation with phenylephrine (1 μmol/L for 96 h) to induce hypertrophy in the presence or absence of exendin-4 (0.1 μmol/L) [33]. H9c2 cardiomyoblast cross-sectional area was quantified by blinded digital image analysis (NIS-Elements) as an index of cell hypertrophy. HL-1 cells were maintained in Claycomb media containing 10 % FCS, 0.1 mmol/L norepinephrine, 2 mmol/L l-glutamate, 100 U/ml penicillin and 100 µg/ml streptomycin. At passage, they were plated and cultured to sub-confluency (70–80 %) prior to incubation with doxorubicin (5 μmol/L for 24 h) to induce apoptosis in the presence or absence of exendin-4 (0.1 μmol/L) [34]. Caspase 3/7 activity (Caspase-Glo^®^, Promega UK) was quantified as a measure of apoptosis and data normalised to cell viability assessed by CellTiter-Blue^®^ assay (Promega, UK).

### Real-time reverse transcription-PCR

Total RNA was extracted from LV homogenate using TRI reagent (Sigma-Aldrich), and cDNA synthesised by reverse transcription (Applied Biosystems). mRNA expression of atrial natriuretic peptide (ANP), procollagen IαI, procollagen IIIαI, connective tissue growth factor (CTGF), fibronectin, TGF-β_3_, IL-10, IL-1β, IL-6 and GLP-1R was analysed by real-time reverse transcription-PCR (RT-PCR) using fluorescent SYBR Green (Prism 7300, Applied Biosystems) and β-actin for normalisation by the comparative *C*_t_ method. Primer sequences are shown in Online Resource 1.

### Western blotting

LV protein was extracted by homogenisation with ice-cold RIPA buffer, as previously described [31], and 20 μg loaded onto a 10 % SDS-PAGE gel before blotting on a polyvinylidene fluoride membrane (Immobilon-FL; Millipore). Membranes were probed overnight at 4 °C with rabbit monoclonal antibodies (1:1000) against total glycogen synthase kinase 3β (GSK3β; Cell Signaling Technology), phospho-GSK3β (Ser9; Cell Signaling Technology) and discoidin domain receptor 2 (DDR2; Abcam), and rabbit polyclonal antibodies (1:1000) against total Akt, phospho-Akt (Ser473), Smad2/3 (both from Cell Signaling Technology) and phospho-Smad2/3 (Ser423/425; Santa Cruz Biotechnology), using rabbit polyclonal HPRT2 antibody (Abcam; 1:1000) as a loading control. This was followed by incubation with peroxidase-labelled goat anti-rabbit secondary antibody (Cell Signaling Technology; 1:10,000) for 60 min at room temperature, before the membrane was developed in a darkroom using ECL reagent (Millipore), scanned and quantified by densitometry (ImageJ).

### Murine cardiac fibroblast isolation and culture

Murine cardiac fibroblasts were isolated from male C57BL/6J mice based on a previously published method [35]. Mice (5 per isolation) were euthanised with sodium pentobarbitone (200 mg/kg i.p.) and heparin (100 IU/100 g i.p.) and their hearts removed immediately into ice-cold Krebs–Henseleit buffer (KHB). After rinsing twice in KHB, ventricles were isolated, finely minced in KHB and mixed vigorously with 4 ml Liberase™ solution (Roche, Sussex, UK) at 37 °C for 8 min. The supernatant was then removed and added to 10 ml ice-cold KHB to inhibit enzyme activity, whilst 3 ml Liberase™ was added to the undigested tissue and the process repeated a 3 further times. The combined supernatant was filtered and centrifuged at 200*g* for 5 min and the resultant pellet resuspended in DMEM supplemented with 10 % FBS, 20 mmol/L l-glutamine, 100 U/ml penicillin and 100 mg/ml streptomycin, and transferred to a 1 % gelatin-coated T75 flask with 10 ml DMEM at 37 °C in a humidified atmosphere of 5 % CO_2_. After 2 h, non-adherent cells were removed and the remaining primary cardiac fibroblasts cultured for approximately 7 days until 90 % confluent. Cells were then washed with PBS, detached with 0.25 % trypsin and centrifuged in DMEM at 200*g* for 5 min. The cell pellet was suspended in fresh DMEM and fibroblasts seeded at 1:2 for expansion before being used for experiments at passage 2. Fibroblasts were treated with or without TGF-β (5 ng/ml), to induce myofibroblast differentiation, in the presence or absence of exendin-4 (10 nmol/L) for 24 h prior to assessment of gene expression. Protein was prepared as described previously, and probed for α-smooth muscle actin using a mouse monoclonal antibody (Dako; 1:2000) and mouse monoclonal β-actin antibody (Cell Signaling Technology; 1:50,000) as a loading control. This was followed by incubation with Alexa Fluor^®^ 594 goat anti-mouse (Molecular Probes; 1:10,000) and horse anti-mouse (Cell Signaling Technology; 1:10,000) secondary antibodies, respectively, for 60 min at room temperature, before membranes were developed in a darkroom using ECL reagent (Millipore), scanned and quantified by densitometry (ImageJ). The same α-smooth muscle actin antibody was used at 1:200 to visualise actin myofilament differentiation with DAPI nuclear counterstaining imaged on a fluorescence microscope at 200x magnification (Nikon Eclipse). RNA/cDNA was also prepared as described previously, and CTGF mRNA expression assessed by real-time RT-PCR using fluorescent SYBR Green and GAPDH for normalisation. Primer sequences are shown in Online Resource 1.

### Murine bone marrow-derived macrophage isolation and culture

Murine bone marrow-derived macrophages were isolated from male C57BL/6J mice, as previously described [36]. Mice were euthanised with sodium pentobarbitone (200 mg/kg i.p.) and their femurs isolated and rinsed in DMEM supplemented with 10 % FBS and 100 U/ml penicillin and 100 mg/ml streptomycin. Bone marrow cells were then flushed out with fresh DMEM using a 23-gauge needle and centrifuged at 500*g* for 10 min at 4 °C. Media was aspirated and cells were resuspended in ACK lysis buffer at room temperature for 3 min to destroy any contaminating red blood cells. The lysate was then quenched with excess DMEM and centrifuged as before. Bone marrow cells were then resuspended in DMEM supplemented with L929 cell supernatant (1:6) containing macrophage colony-stimulating factor to induce differentiation, and seeded into a 150 mm tissue culture plate. At day 2, media was replaced with fresh DMEM supplemented with L929 supernatant to remove any non-adherent cells. At day 4, cells were washed and scraped into DMEM prior to centrifugation at 500*g* for 10 min at 4 °C, and seeded 1:3 into tissue culture plates containing DMEM supplemented with L929 supernatant. At day 8, cells were counted and split into 6-well plates, each containing 1 million cells, prior to incubation for a further 24 h in DMEM without L929 supernatant in the presence or absence of exendin-4 (1 nmol/L). RNA/cDNA and protein were then prepared for gene expression analysis, as described previously. mRNA expression of CD11b, IL-10, MMP-9, FGF-2, IL-1β and CCL2 was analysed by real-time RT-PCR using fluorescent SYBR Green and GAPDH for normalisation. Primer sequences are shown in Online Resource 1. Secreted cytokine expression was assessed in pooled conditioned media from each experimental group using a Proteome Profiler™ antibody array (R&D Systems). Immunocytochemistry for GLP-1R was performed in both BMDM and cardiac fibroblasts using a rabbit polyclonal antibody (Abcam: 1:200) with DAPI nuclear counterstaining, whilst GLP-1R protein expression was quantified in BMDM by Western blot using the same primary antibody (1:1000) and a mouse monoclonal β-actin antibody (Cell Signaling Technology: 1:50,000) as the loading control.

### Statistics

Data are expressed as mean ± SEM and were analysed by either an unpaired *t* test, one-way ANOVA followed by a Bonferroni or Dunnett’s multiple comparison test, or Kaplan–Meier survival analysis using a log-rank test. *P* < 0.05 was considered to be statistically significant.

## Results

### Metabolic data

Metabolic parameters are summarised in Table 1. Body weight was not different between groups. Similarly, chronic infusion with exendin-4 for 4 weeks had no effect on plasma glucose, HbA1c, insulin, cholesterol or triglyceride concentrations, whilst minimal differences in plasma adipokine expression were observed (Online Resource 2).

**Table 1:** Effect of exendin-4 on metabolic parameters

	Sham control	MI control	Sham exendin-4	MI exendin-4
Body weight (g) *n* = 19	22.9 ± 0.4	23.7 ± 0.5	23.0 ± 0.3	23.2 ± 0.3
Plasma glucose (mmol/L) *n* = 7–12	13.6 ± 1.8	12.6 ± 0.7	13.1 ± 1.0	13.9 ± 0.9
Plasma HbA1c (%) *n* = 4–6	6.81 ± 0.23	6.90 ± 0.43	6.32 ± 0.32	6.08 ± 0.22
Plasma insulin (mmol/L) *n* = 10–12	0.56 ± 0.06	0.47 ± 0.04	0.53 ± 0.07	0.64 ± 0.11
Plasma cholesterol (mmol/L) *n* = 10–12	1.71 ± 0.07	1.88 ± 0.08	1.85 ± 0.09	1.84 ± 0.10
Plasma triglyceride (mmol/L) *n* = 10–11	1.32 ± 0.11	1.24 ± 0.14	0.90 ± 0.09	1.25 ± 0.11

Mean ± SEM. *P* = NS

### Infarct size

Area at risk was similar between MI groups (control: 73.6 ± 2.0, exendin-4: 72.4 ± 2.3 % LV; *n* ≥ 11) after 4 weeks. Infarct sizes were also comparable between MI groups (control: 57.9 ± 4.2, exendin-4: 59.2 ± 4.3 % area at risk; *n* ≥ 11). However, survival rate at 4 weeks post-MI tended to be higher after exendin-4 treatment (MI control: 67.6 %, *n* = 68; MI exendin-4: 80.3 %, *n* = 76; *P* = 0.07). Only one death was observed in the sham groups (*n* = 58).

### Cardiac function

**Fig. 1:**
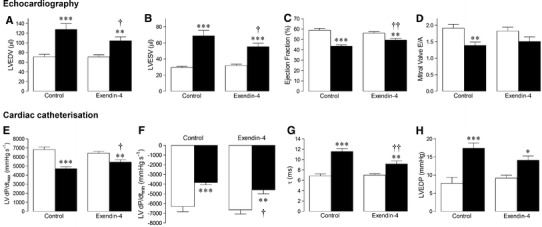
Effect of exendin-4 on cardiac function post-MI. **a** LV end-diastolic volume (LVEDV), **b** LV end-systolic volume (LVESV), **c** LV ejection fraction, **d** mitral valve (MV) *E*/*A* ratio, assessed by echocardiography (*n* = 10–16). **e** LV d*P*/d*t*_max_; **f** LV d*P*/d*t*_min_; **g** *τ*; **h** LV end-diastolic pressure (LVEDP), assessed by cardiac catheterisation (*n* = 9–15). *White columns* sham, *black columns* MI, mean ± SEM. *P < 0.05, **P < 0.01, ***P < 0.001, versus corresponding sham; ^†^ *P* < 0.05, ^††^ *P* < 0.01, MI exendin-4 versus MI control

Echocardiography data are presented in Fig. 1a–d. LV dilatation in response to MI, as measured by LVEDV, was reduced by exendin-4. Similarly, exendin-4-treated mice demonstrated improved systolic function post-MI, reflected by higher ejection fraction and lower LVESV. Furthermore, diastolic dysfunction, indicated by a reduced mitral valve *E*/*A* ratio in MI controls, was not evident after exendin-4 treatment. Similar functional changes were observed after assessment of LV dimension-derived parameters (LVEDD, LVESD, fractional shortening) and these data are presented in Online Resource 3. Heart rate remained similar between groups.

Cardiac catheterisation data are summarised in Fig. 1e–h. Post-MI decreases in systolic function, measured by LVd*P*/d*t*_max_, were attenuated by exendin-4. Similarly, exendin-4-treated mice demonstrated better preserved LV relaxation after MI, indexed by LVd*P*/d*t*_min_ and the isovolumetric relaxation time constant, *τ*, and a tendency towards improved diastolic function, reflected by reduced LV end-diastolic pressure. Mean arterial blood pressure (control: 64.2 ± 0.9, exendin-4: 67.4 ± 1.2, MI control: 64 ± 2.7, 66.0 ± 1.6 mmHg; *n* = 9–15, *P* = NS) and heart rate (control: 484 ± 24, exendin-4: 486 ± 16, MI control: 478 ± 14, 472 ± 13 bpm; *n* = 9–15, *P* = NS) remained similar between groups.

### Cardiomyocyte remodelling

**Fig. 2:**
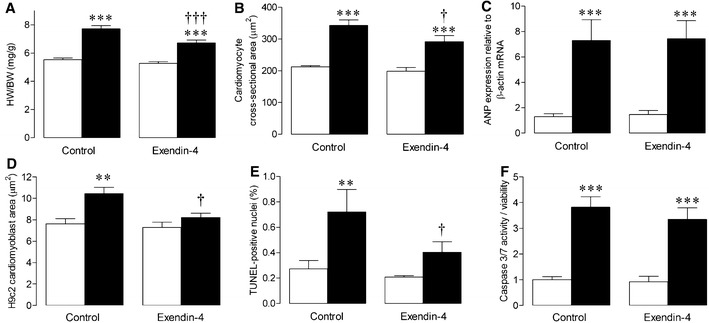
Effect of exendin-4 on cardiomyocyte remodelling after MI. **a** Heart weight/body weight (HW/BW; *n* = 24–29), **b** cardiomyocyte cross-sectional area (*n* = 6), **c** ANP mRNA expression (*n* = 6–8), **d** phenylephrine-induced H9c2 cardiomyoblast hypertrophy (*n* = 7–8), **e** TUNEL staining (*n* = 6), **f** doxorubicin-induced HL-1 cardiomyocyte apoptosis (*n* = 6). *White columns* sham (**a**–**c**, **e**) or untreated cells (**d**, **f**); *black columns* MI (**a**–**c**, **e**) or phenylephrine/doxorubicin-treated cells (**d**, **f**); mean ± SEM. **P < 0.01, ***P < 0.001, versus corresponding sham/untreated control; ^†^ *P* < 0.05, ^†††^ *P* < 0.001, MI exendin-4 versus MI control or phenylephrine exendin-4 versus phenylephrine control

Chronic exendin-4 treatment reduced MI-induced myocardial hypertrophy, as indicated by heart/body weight ratio and cardiomyocyte area (Fig. 2a, b), although it had no effect on mRNA expression of ANP (Fig. 2c), an established marker of hypertrophy. Similarly, in vitro phenylephrine-induced hypertrophy of H9c2 cardiomyoblasts was attenuated by exendin-4 (Fig. 2d). MI-induced cardiomyocyte apoptosis, assessed in LV sections by TUNEL staining, was also decreased by exendin-4 (Fig. 2e), although acute treatment had no significant effect on in vitro doxorubicin-induced apoptosis in HL-1 cardiomyocytes (Fig. 2f).

### Extracellular matrix remodelling

**Fig. 3:**
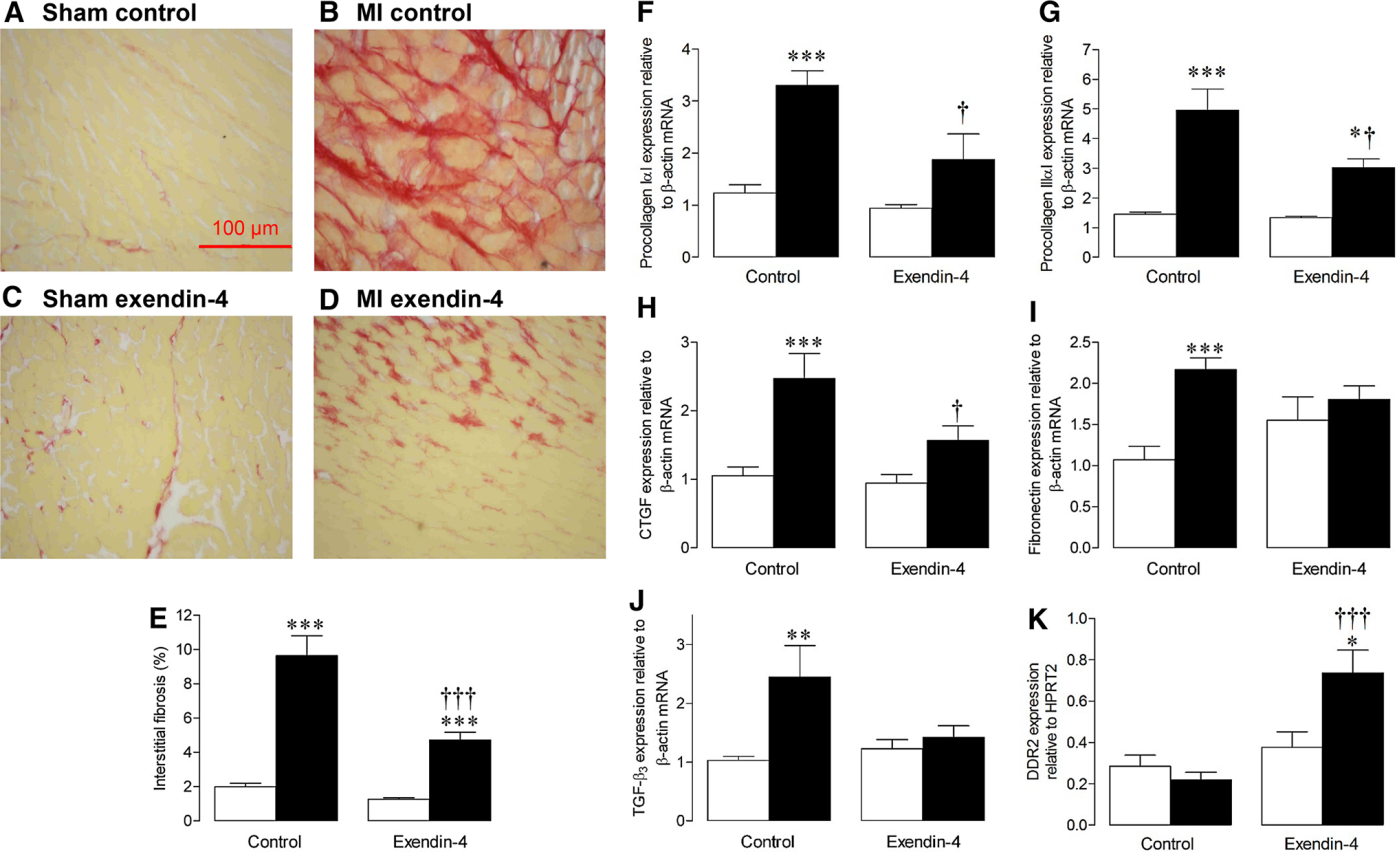
Effect of exendin-4 on ECM remodelling after MI. **a**–**d** Representative LV sections stained with picrosirius red to assess interstitial fibrosis, **e** quantification data (*n* = 6). **f**–**j** mRNA expression of ECM genes by real-time RT-PCR (*n* = 5–12), **k** DDR2 protein expression by Western blot (*n* = 6). *White* *columns* sham; *black* *columns* MI; mean ± SEM. *P < 0.05, **P < 0.01, ***P < 0.001, versus corresponding sham; ^†^ *P* < 0.05, ^†††^ *P* < 0.001, MI exendin-4 versus MI control

Interstitial fibrosis was markedly decreased in exendin-4-treated MI mice compared to MI controls (Fig. 3a–e). Consistent with this, MI-induced increases in mRNA expression of several key ECM genes, procollagen IαI, procollagen IIIαI, CTGF, fibronectin and TGF-β_3_, were reduced by exendin-4 (Fig. 3f–j). In addition, protein expression of the fibroblast marker, DDR2, was increased in MI exendin-4-treated mice compared to MI controls (Fig. 3k) [37, 38].

### Myocardial inflammation

**Fig. 4:**
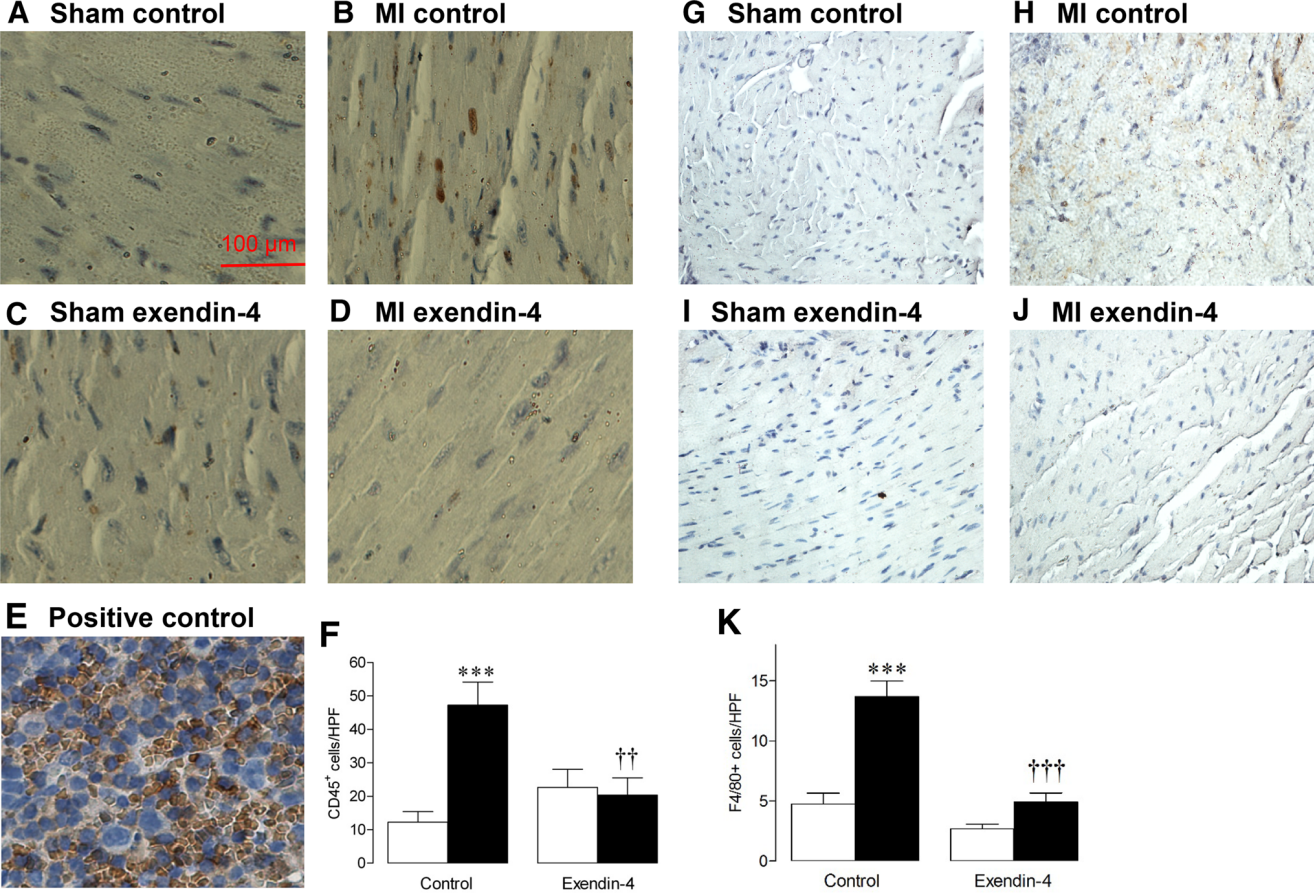
Effect of exendin-4 on myocardial inflammation after MI. Representative LV sections stained for **a**–**e** CD45, with positive spleen control, and **g**–**j** F4/80, to assess leukocyte and macrophage infiltration, respectively. **f**, **k** Quantification data (*n* = 5–6). *White columns* sham; *black columns* MI; mean ± SEM. ***P < 0.001, versus corresponding sham; ^††^ *P* < 0.01, ^†††^ *P* < 0.001, MI exendin-4 versus MI control

Myocardial infiltration of CD45-positive leukocytes and F4/80-positive macrophages after MI was completely prevented by exendin-4 (Fig. 4a–k). Consistent with this, MI-induced alterations in mRNA expression of the inflammatory cytokines, IL-10, IL-1β and IL-6, were normalised by exendin-4 (Online Resource 4).

### GLP-1R receptor expression

GLP-1R mRNA expression was increased in MI controls and attenuated by exendin-4 (Online Resource 5).

### Investigation of potential cardioprotective signalling mechanisms

**Fig. 5:**
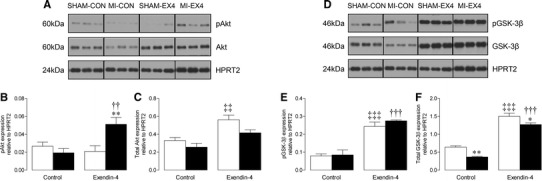
Effect of exendin-4 on Akt and GSK3β expression after MI. Representative Western blots for total and phosphorylated **a** Akt, and **d** GSK-3β; blots were stripped and reprobed and normalised to HPRT2, which is displayed within each *panel* for comparison. **b**, **c**, **e**, **f** Quantification data (*n* = 6). *White columns* sham; *black columns* MI; mean ± SEM. *P < 0.05, **P < 0.01, versus corresponding sham; ^††^ *P* < 0.01, ^†††^ *P* < 0.001, MI exendin-4 versus MI control; ^‡‡^ *P* < 0.01, ^‡‡‡^ *P* < 0.001, sham exendin-4 versus sham control

**Fig. 6:**
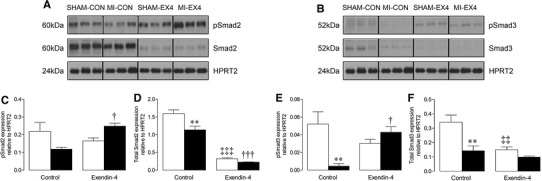
Effect of exendin-4 on Smad2/3 expression after MI. Representative Western blots for total and phosphorylated **a** Smad2, and **d** Smad3; blots were stripped and reprobed and normalised to HPRT2, which is displayed within each *panel* for comparison. **b**–**c**, **e**–**f** Quantification data (*n* = 6). *White* *columns* sham; *black* *columns* MI; mean ± SEM. **P < 0.01, versus corresponding sham; ^†^ *P* < 0.05, ^†††^ *P* < 0.001, MI exendin-4 versus MI control; ^‡‡^ *P* < 0.01, ^‡‡‡^ *P* < 0.001, sham exendin-4 versus sham control

Several candidate pathways which may mediate the protective effects of exendin-4 on post-MI remodelling were investigated. Phosphorylation of the pro-survival kinase Akt was clearly elevated by exendin-4 after MI, in association with an apparent increase in total protein expression (Fig. 5a–c). Furthermore, both total and phosphorylated GSK-3β, a known substrate of Akt, which has been proposed as a convergence point for several cardioprotective pathways and specifically suppresses cardiac fibroblast-driven remodelling [39, 40], was markedly increased by exendin-4 (Fig. 5d–f), indicating deactivation of GSK-3β signalling. In addition, the expression and activity of two key Smad proteins, which are important regulators of TGF-β signalling and ECM turnover with documented cardioprotective actions [41, 42], were specifically modulated by exendin-4 post-MI. MI-induced decreases in both total and phosphorylated Smad2 and Smad3 were prevented by exendin-4, although the drug itself also reduced total protein expression in shams (Fig. 6a–f).

### Cell studies

**Fig. 7:**
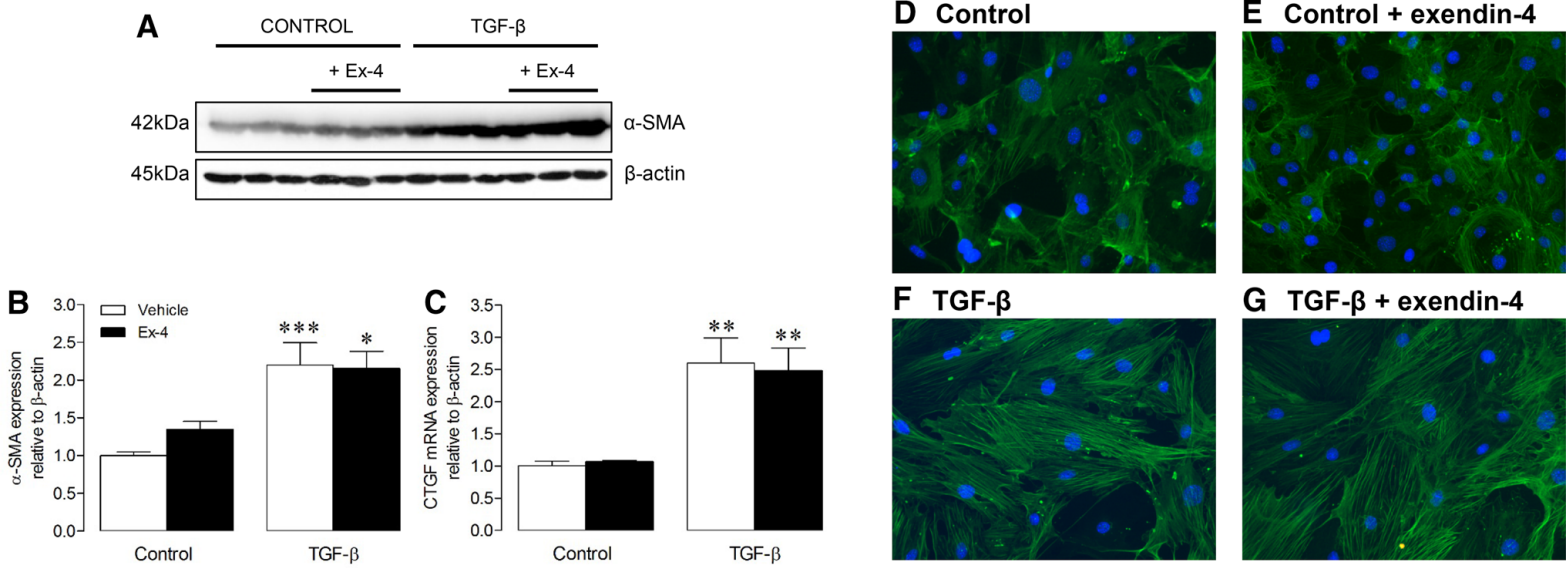
Effect of exendin-4 on isolated murine cardiac fibroblasts. **a** Representative Western blot for α-SMA in cardiac fibroblasts treated with/without TGF-β (5 ng/ml) in the presence/absence of exendin-4 (Ex-4; 10 nmol/L) for 24 h; blots were stripped and reprobed and normalised to β-actin. **b** α-SMA protein quantification data (*n* = 8). **c** CTGF mRNA expression in cardiac fibroblasts by real-time RT-PCR (*n* = 6). **d**–**g** Representative images of cardiac fibroblasts stained for α-SMA to visualise myofibroblast differentiation with DAPI nuclear counterstaining. Data are mean ± SEM. *P < 0.05, **P < 0.01; ***P < 0.001, versus corresponding control

**Fig. 8:**
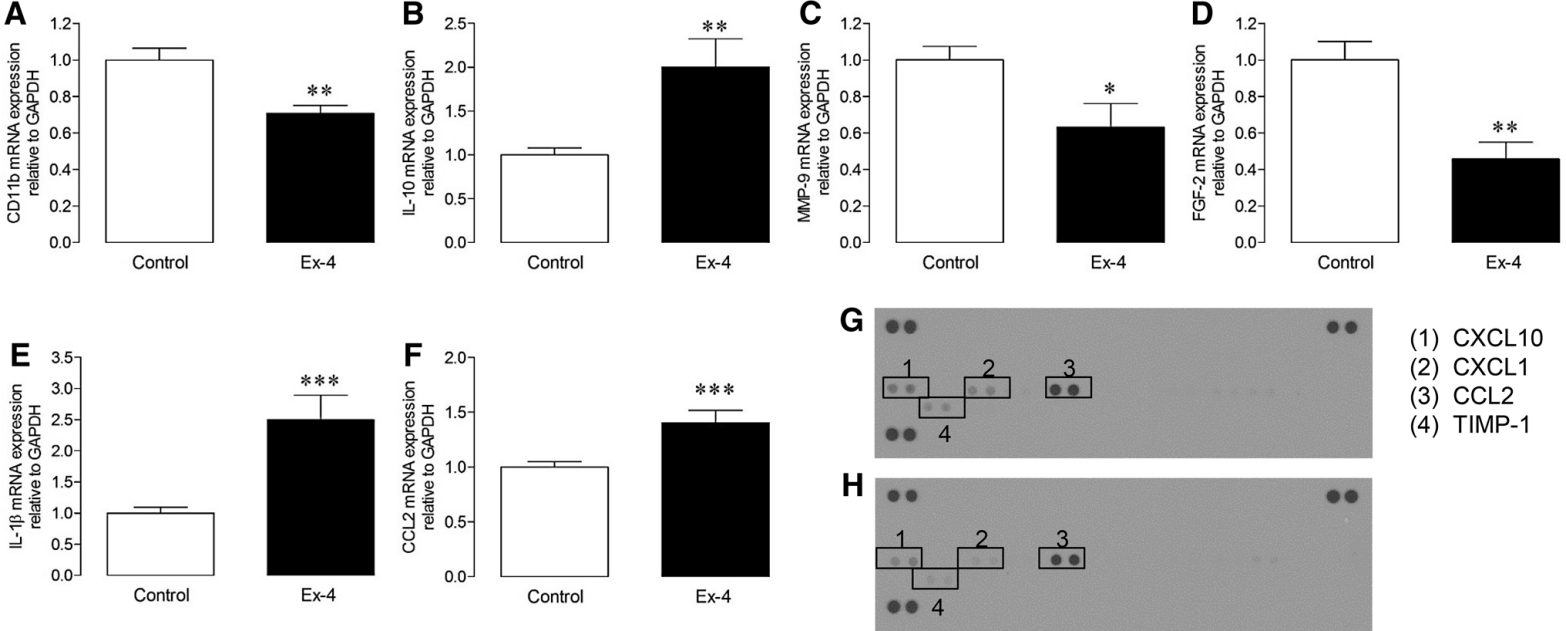
Effect of exendin-4 on inflammatory gene expression in isolated BMDM. **a**–**f** Cellular mRNA expression by real-time RT-PCR (*n* = 6–8), and **g**, **h** conditioned media cytokine protein array blots, of macrophage response genes from BMDM treated with/without exendin-4 (Ex-4; 1 nmol/L) for 24 h. Data are mean ± SEM. *P < 0.05, **P < 0.01; ***P < 0.001, versus corresponding control

To investigate cell-specific effects of exendin-4 relevant to post-MI remodelling, a series of mechanistic studies were conducted in murine cardiac fibroblasts and BMDM. Exendin-4 had no effect on basal and TGF-β-stimulated α-SMA and CTGF expression in cardiac fibroblasts (Fig. 7a–c). In addition, actin myofilament density, visualised by α-SMA staining, was similar between fibroblasts treated with/without TGF-β in the presence/absence of exendin-4 (Fig. 7d–g), confirming that exendin-4 does not appear to exert direct effects on these cells. Further studies confirmed previous reports that cardiac fibroblasts do not express the GLP-1R [8], whilst significant GLP-1R expression was detected in BMDM which appeared to be increased by exendin-4 (Online Resource 6). Notably, treatment of BMDM with exendin-4 for 24 h resulted in modulation of several macrophage response genes relevant to tissue repair/remodelling. Cellular mRNA expression of CD11b, MMP-9 and FGF-2 was reduced with that of IL-10, IL-1-β, and CCL2 increased (Fig. 8a–f), whilst levels of key secreted proteins (CXCL10, CXCL1, CCL2, TIMP-1) were decreased by exendin-4 (Fig. 8g, h).

## Discussion

Here, it is reported for the first time that chronic GLP-1R activation post-MI protects against adverse ventricular remodelling via specific actions on individual remodelling components which appear to involve preferential targeting of inflammation and the ECM. This study clearly demonstrates that exendin-4 protects against cardiac contractile dysfunction after experimental MI, which is associated with reduced cardiomyocyte hypertrophy/apoptosis, and marked attenuation of myocardial inflammation and ECM remodelling. Importantly, these beneficial effects on post-MI remodelling occurred independently of the established glycaemic and acute cardioprotective actions of GLP-1 and were associated with modulation of both Akt/GSK-3β and Smad2/3 signalling and improved survival. Furthermore, complementary cell studies found that exendin-4 modulated macrophage response gene expression and protein secretion, but had no effect on differentiation of cardiac fibroblasts, which do not express the GLP-1R, suggesting that the observed actions on ECM remodelling may occur secondary to modulation of myocardial inflammation, although it is likely that cardiomyocyte signalling pathways are also involved.

The rationale for this investigation was based on several studies indicating that GLP-1R activation confers protection against acute ischaemic damage [8, 19, 20] and two subsequent reports that GLP-1 analogues administered 2 days and 2 weeks post-MI improve survival and cardiac structure/function [23, 43]. Here, we chose to employ an alternate treatment regimen of continuous exendin-4 infusion starting immediately after MI or sham surgery to specifically assess effects on distinct aspects of post-MI remodelling independently of GLP-1R-mediated alterations in acute infarct remodelling. Indeed, area at risk and infarct size measurements were comparable between groups indicating that the observed effects were likely to be due to direct actions on post-MI ventricular remodelling. Importantly, the concentration of exendin-4 employed in our study was comparable to those commonly used in previous rodent studies relating to the anti-diabetic and cardioprotective actions of GLP-1 [12, 15, 16, 32]. Furthermore, our studies were performed in wild-type mice, in which body weight and circulating glucose, insulin and lipids were unaltered by exendin-4, together with minimal changes in adipokine expression, suggesting that the reported cardiac effects are likely to be due to direct actions of exendin-4, although it is impossible to completely exclude peripheral effects.

Several previous studies have indicated that GLP-1 modulates basal cardiac function and protects against contractile dysfunction associated with both experimental and clinical CHF [10, 13, 14, 16, 18, 21, 22]. Indeed in our study, the development of MI-induced systolic dysfunction (increased LVESV, decreased ejection fraction and LVd*P*d*t*_max_) was attenuated by exendin-4. Chronic treatment with exendin-4 also improved LV relaxation after MI (increased LVd*P*/d*t*_min_, reduced *τ*) and protected against diastolic dysfunction (preserved mitral valve *E*/*A* ratio, reduced LV end-diastolic pressure). Furthermore, LV dilatation (measured by LVEDD and LVEDV) observed in MI control mice was reduced by exendin-4.

It seems reasonable to conclude that the marked attenuation of cardiac contractile dysfunction by exendin-4 post-MI occurred secondary to beneficial actions on both the cardiomyocyte and ECM, which are established as key remodelling components in this setting [3]. Indeed, exendin-4 caused modest reductions in morphometric heart/body weight and cardiomyocyte cross-sectional area after MI, together with reduced in vivo cardiomyocyte apoptosis. Acute exendin-4 treatment also inhibited in vitro phenylephrine-induced H9c2 cardiomyoblast hypertrophy but failed to reduce doxorubicin-induced apoptosis of HL-1 cardiomyocytes, assessed by caspase 3/7 activation. However, it is important to highlight that our conducted assessment of cardiomyocyte apoptosis was minimal as only a single output was measured in both ventricular tissue and cultured cells which represents a significant limitation. Nonetheless, other studies have consistently shown GLP-1 to protect cardiomyocytes from apoptosis both in vivo and in vitro [15, 30], although reported effects on cardiomyocyte hypertrophy have been varied. Whilst some have reported the DPP-4 inhibitor, sitagliptin, and exendin-4 to have no effect on cardiomyocyte hypertrophy in experimental MI and type 1 diabetes, respectively, others have shown that GLP-1R activation with liraglutide reduces cardiomyocyte hypertrophy after both MI and high-fat feeding [15, 44–46]. Therefore, it is maybe not surprising that exendin-4 exerted only modest effects on post-MI cardiomyocyte remodelling, which may have occurred directly or secondary to more pronounced effects on the ECM.

Indeed, contractile function is particularly sensitive to ECM remodelling, and exendin-4 markedly reduced MI-induced increases in interstitial fibrosis and ECM gene expression, which is likely to significantly contribute to the associated improvement of LV relaxation (LVd*P*/d*t*_min_, *τ*) and tendency towards increased diastolic function (mitral valve *E*/*A* ratio, LV end-diastolic pressure). Interestingly, this was associated with marked increases in myocardial expression of DDR2 in MI exendin-4 animals, which is an established fibroblast marker also known to modulate collagen biosynthesis [37, 38]. As far as we are aware, these data provide the first direct evidence that GLP-1R activation modulates ECM remodelling, a finding which may have important implications for the use of GLP-1-based therapies in diabetic patients, who are frequently characterised by a disproportionate accumulation of collagen within the heart [47]. Interestingly, our complementary studies in cardiac fibroblasts found that exendin-4 had no effect on TGF-β-induced cell differentiation, which is maybe not surprising as cardiac fibroblasts are known not to express the GLP-1R [8], and suggests that the observed effects on ECM remodelling may be mediated via another cell type. Indeed, we found that myocardial GLP-1R expression was increased after MI (which may represent an adaptive response) and was normalised by exendin-4, although it is also possible that the mechanism may involve established GLP-1R-independent pathways [8, 48].

The most likely remodelling process by which exendin-4 may mediate indirect effects on the cardiac fibroblast would appear to be inflammation, which is becoming increasingly recognised as an important mediator of post-MI remodelling capable of exerting specific regulatory actions on several remodelling components, but which preferentially targets the ECM [43, 49]. Indeed, in this study, experimental MI was associated with marked myocardial infiltration of CD45^+^ leukocytes and F4/80^+^ macrophages and altered expression of inflammatory cytokines, IL-10 (known regulator of tissue remodelling), IL-1β and IL-6 (both critical for fibroblast differentiation) [50, 51]. Importantly, exendin-4 completely prevented these changes suggesting that the observed benefits on post-MI remodelling may have occurred, at least partly, secondary to modulation of inflammation, which is consistent with previous reports of anti-inflammatory actions in other organs, such as the pancreas [29]. Furthermore, complementary experiments in BMDM, which expressed the GLP-1R, found that exendin-4 differentially modulated basal levels of both resident and secreted macrophage response genes, which are known to contribute to tissue remodelling and repair [52, 53]. Exendin-4 decreased macrophage expression of both CD11b, which plays a key role in monocyte adhesion, and MMP-9 and FGF-2 which are important drivers of ECM remodelling, whilst tissue-protective IL-10 and pro-inflammatory IL-1β and CCL2 were upregulated. Furthermore, exendin-4 reduced levels of secreted CXCL10, CXCL1, CCL2 and TIMP-1, all of which are key modulators of macrophage function [49]. Taken together, these data clearly suggest that the observed effects of exendin-4 on ECM remodelling are mediated, at least partly via indirect macrophage-driven actions on the cardiac fibroblast. However, the underlying mechanisms are likely to be complex and require detailed further investigation.

As previous studies focusing on the acute cardioprotective actions of GLP-1 have consistently demonstrated modulation of both Akt and GSK-3β [15, 16, 20], we chose to investigate effects of exendin-4 on this pathway in the setting of post-MI remodelling. Indeed, in our study, Akt and also GSK-3β, which has been reported to specifically suppress cardiac fibroblast-mediated remodelling [39], were upregulated by exendin-4 after MI, at both total and phosphorylated protein level, suggesting that modulation of cardiomyocyte Akt/GSK-3β signalling plays a key role in mediating the chronic cardioprotective actions of GLP-1R activation and may thereby indirectly influence ECM remodelling. Notably, chronic cardiomyocyte-specific Akt activation has recently been shown to promote both pathological hypertrophy and fibrosis [54], supporting the suggestion that exendin-4 induced modulation of cardiomyocyte Akt/GSK-3β signalling may contribute to the observed ECM effects. In addition to its effects on Akt/GSK-3β signalling, exendin-4 prevented MI-induced decreases in phosphorylated Smad2 and Smad3, indicating that it may promote activity of these two key Smad proteins in the viable myocardium post-MI. Although Smad2 and Smad3 signalling are closely linked [40], their precise role in cardiac remodelling is unknown. For example, some studies indicate that they may exert divergent cardiac actions, with Smad3 being pro-fibrotic and Smad2 protective against cardiomyocyte remodelling, whereas others suggest that cardiac fibrosis is promoted by Smad2 and inhibited by Smad3 [39, 41, 55, 56]. Interestingly, other groups have also questioned the dependence of cardiac Smad signalling on TGF-β [57, 58]. Therefore, although it appears that exendin-4 exerts its benefits on post-MI remodelling, at least partly via activation of Smad2/3, the precise nature of these actions remains unclear.

Although our data clearly suggest that exendin-4 exerts distinct protective actions on post-MI remodelling via apparently preferential modulation of myocardial inflammation and the ECM, it is important to consider several limitations. A major issue with such studies is the inherent risk of selection bias as only surviving animals are studied, so it is possible that infarcts may have been larger in MI controls thereby explaining their tendency towards increased mortality. However, MI animals generally died within 7 days due to cardiac rupture, as opposed to acute heart failure, which is more likely to reflect impaired wound healing, and further to our 4-week data, may indeed be expected to be improved by exendin-4. In addition, as exendin-4 was infused immediately post-MI, it seems unlikely that this treatment would affect area at risk and extent of infarction. In this regard, it should be noted that sample numbers were not consistently lower in MI control versus exendin-4 treated animals and that any disparities were due to practical or technical issues. There are also potential limitations with our experimental model. Firstly, while there is a general argument that both sexes should be included in disease investigation, only female mice were assessed here due to animal welfare considerations. Indeed, we have previously employed female mice for post-MI remodelling studies as have several other leading groups [31, 59, 60]. Secondly, we chose to induce permanent coronary artery ligation and so the influence of reperfusion could not be studied. Thirdly, we used a supra-clinical dose of exendin-4 to identify specific remodelling actions and effects of lower therapeutic levels were not assessed. Furthermore, as our initial aim was to precisely define the actions of exendin-4 on post-MI remodelling by focussing on individual components, no primary end point was selected. It is also important to recognise that the use of cell lines to interrogate in vitro effects of exendin-4 on cardiomyocyte hypertrophy may have limited relevance to the in vivo setting, although they may still provide useful complementary data. Nonetheless, our findings indicate that exendin-4 protects against post-MI remodelling and promotes preferential modulation of the ECM. However, although our in vivo data show clear differences in myocardial inflammation and our in vitro data suggest that macrophage signalling may be a potential target for exendin-4 in this setting, we cannot conclude that the observed ECM effects are specifically mediated via macrophage/fibroblast interaction without more sophisticated in vitro and in vivo studies. Similarly, the effects of exendin-4 on cardiomyocyte signalling pathways need to be further interrogated to establish their precise influence on post-MI remodelling.

Despite these limitations, our data strongly suggest that exendin-4 exerts distinct protective actions on post-MI remodelling, independent of its established metabolic and glycaemic effects, which appear to be mediated via preferential modulation of myocardial inflammation and the ECM. Whilst noting that cardiomyocyte signalling pathways are also altered by exendin-4 and so are likely to directly or indirectly influence post-MI remodelling, our findings clearly indicate that exendin-4 exerts distinct actions on specific aspects of post-MI remodelling, and together with previous studies reporting both acute and chronic cardioprotective effects, highlight the potential of GLP-1-based therapeutic strategies. In this regard, it should be noted that the first large-scale GLP-1 cardiovascular trials recently reported that the DPP-4 inhibitors, saxagliptin and alogliptin, did not confer cardiovascular benefit in type 2 diabetes [61, 62], although there are currently no data from long-term randomised clinical trials with GLP-1R agonists, with several ongoing [7]. Although circulating GLP-1 levels in patients treated with DPP-4 inhibitors are much lower than those achieved using GLP-1R agonists, the seemingly disappointing results of these recent trials would appear to support our suggestion that a more selective approach targeted towards specific post-MI remodelling components may be required to realise the clear potential of GLP-1-based strategies in this setting.

## Electronic supplementary material

Below is the link to the electronic supplementary material.

Supplementary material 1 (DOCX 723 kb)
