# Supplementary material for: Exendin-4 protects against post-myocardial infarction remodelling via specific actions on inflammation and the extracellular matrix
Source: Basic Res Cardiol. 2015 Mar 1;110(2):20. doi: 10.1007/s00395-015-0476-7 (PMC4344953; doi:10.1007/s00395-015-0476-7)
Supplement: Supplementary file 1 — Supplementary material 1 (DOCX 723 kb) [file 395_2015_476_MOESM1_ESM.docx]

**SUPPLEMENTARY MATERIAL**

**EXENDIN-4 PROTECTS AGAINST POST-MYOCARDIAL INFARCTION REMODELLING VIA SPECIFIC ACTIONS ON INFLAMMATION AND THE EXTRACELLULAR MATRIX**

Emma Robinson^1*^, Roslyn S. Cassidy^1*^, Mitchel Tate^1*^, Youyou Zhao^1^, Samuel Lockhart^1^, Danielle Calderwood^2^, Rachel Church^1^, Mary K. McGahon^1^, Derek P. Brazil^1^,

Barbara J. McDermott^1^, Brian D. Green^2^, David J. Grieve^1^

^*^These three authors contributed equally to this work.

Queen’s University Belfast, ^1^Centre for Experimental Medicine, Belfast BT12 6BA, UK and ^2^School of Biological Sciences, Belfast BT9 5AG, UK

**ONLINE RESOURCE 1:** Real-time RT-PCR primer sequences

| Gene | Primer | Sequence |
| --- | --- | --- |
| ANP  β-actin  CTGF  Fibronectin  Procollagen IαI  Procollagen IIIaI  TGF-β_3_  IL-1β  IL-6 | Forward  Reverse  Forward  Reverse  Forward  Reverse  Forward  Reverse  Forward  Reverse  Forward  Reverse  Forward  Reverse  Forward  Reverse  Forward | 5'- CGTGCCCCGACCCACGCCAGCATGGGCTCC-3'  5'- GGCTCCGAGGGCCAGCGAGCAGAGCCCTCA-3'  5’-CGTGAAAAGATGACCCAGATCA-3’  5’-TGGTACGACCAGAGGCATACAG-3’  5’-GCTGCCTACCGACTGGAAGAC-3’  5’-CCTAATGGCTTCCACCCTCTTC-3’  5’-CCGGTGGCTGTCAGTCAGA-3’  5’-CCGTTCCCACTGCTGATTTATC-3’  5’-CCTCAGGGTATTGCTGGACAAC-3’  5’-TTGATCCAGAAGGACCTTGTTTG-3’  5’-AGGAGCCAGTGGCCATAATG-3’  5’-TGACCATCTGATCCAGGGTTTC-3’  5’-GGAGAGAGTCCAACTGGGTCTG-3’  5’-ACATTTTCCAGTATGTCTCCATTGG-3’  5’-TGTGGCTGTGGAGAAGCTGT-3’  5’-CAGCTCATATGGGTCCGAGA-3’  5’-CACGGCCTTCCCTACTTCAC-3’ |
|  | Reverse | 5’-TGCAAGTGCATCATCGTTGT-3’ |
| CD11b  IL-10 | Forward  Reverse  Forward  Reverse | 5’-AAACCACAGTCCCGCAGAGA-3’  5’-CGTGTTCACCAGCTGGCTTA-3’  5’-TGCAGGACTTTAAGGGTTACTTGG-3’  5’-GGCCTTGTAGACACCTTGGTC-3’ |
| MMP-9 | Forward  Reverse | 5’-CAAGTGGGACCATCATAACATCA-3’  5’-GTCTCGCGGCAAGTCTTCA-3’ |
| FGF-2  CCL2  GLP-1R | Forward  Reverse  Forward  Reverse  Forward | 5’-AAGAGCGACCCACACGTCAAAC-3’  5’-GTAACACACTTAGAAGCCAGCAGCC-3’  5’-AGGTCCCTGTCATGCTTCTG-3’  5’-TCTGGACCCATTCCTTCTTG-3’  5’-CATCCACCTGAACCTGTTTGC-3’ |
|  | Reverse | 5’-GGGCAGCGTCTTTGATGAA-3’ |
| GAPDH | Commercial assay obtained from Primer Design Ltd (Southampton, UK)  Catalogue number: SY-mo-600; sequence not available | |

**ONLINE RESOURCE 2**

**
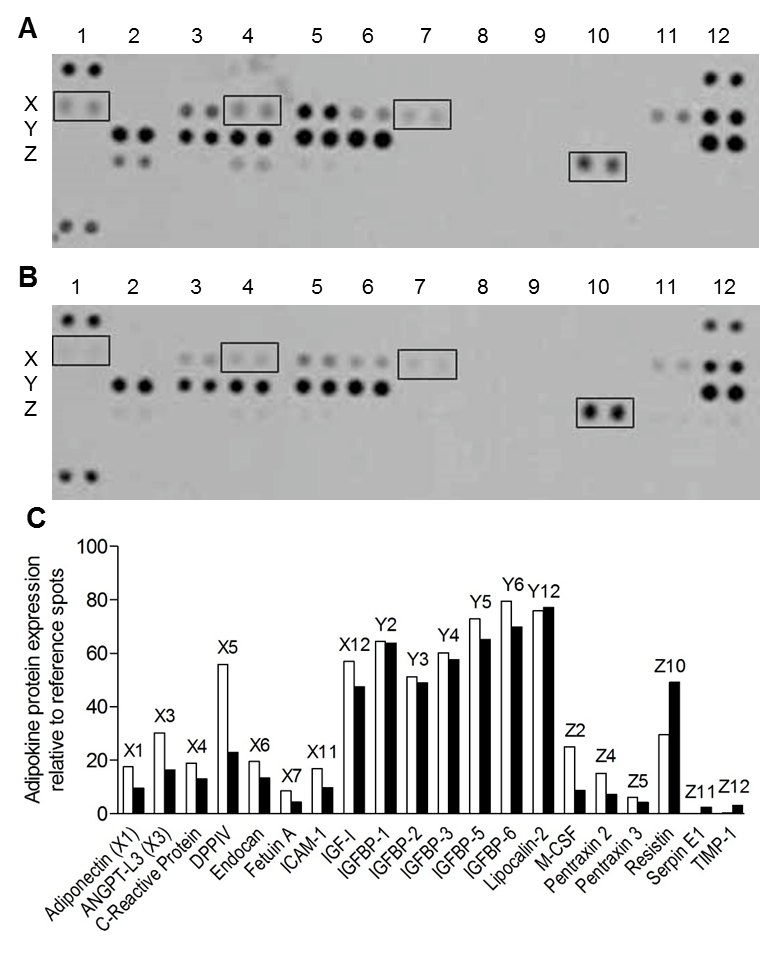
**

Plasma adipokine array blots from (A) MI control and (B) MI exendin-4, with (C) quantification of protein expression; the boxes in panels A and B signify highly abundant proteins which were probed using 10% plasma sample volume. White columns, MI control; black columns, MI exendin-4; pooled sample from 6 animals.

**ONLINE RESOURCE 3**

|  | Sham control | MI control | Sham exendin-4 | MI exendin-4 |
| --- | --- | --- | --- | --- |
| *n* | 16 | 10 | 16 | 10 |
| Heart rate (bpm) | 499±16 | 531±15 | 483±11 | 485±15 |
| LVEDD (mm) | 3.91±0.13 | 5.07±0.18^***^ | 4.00±0.10 | 4.67±0.15^**†^ |
| LVESD (mm) | 2.84±0.08 | 3.84±0.15^***^ | 2.91±0.09 | 3.57±0.12^***^ |
| Fractional Shortening (%) | 30.9±1.4 | 22.1±0.7^***^ | 29.1±1.0 | 24.3±1.1^**^ |
|  |  |  |  |  |

Echocardiographic LV dimension-derived functional parameters in MI and sham mice treated with or without exendin-4. LVEDD, LV end-diastolic diameter; LVESD, LV end-systolic diameter. Mean±SEM. ^**^P<0.01, ^***^P<0.001, versus corresponding sham; ^†^P<0.05, MI exendin-4 versus MI control.

**ONLINE RESOURCE 4**

**
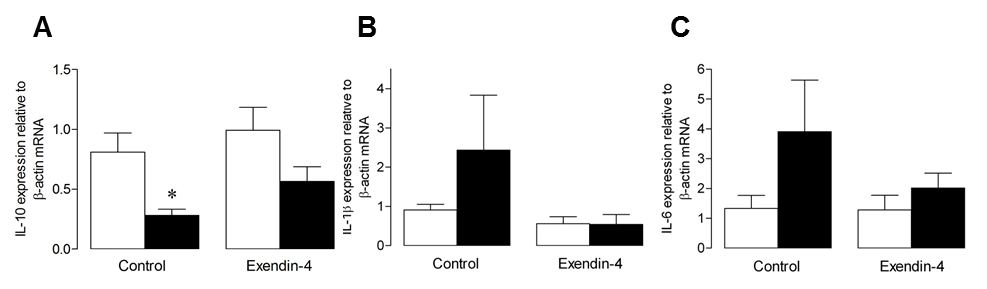
**

Effect of exendin-4 on myocardial inflammatory gene expression after MI. Graph shows mRNA expression of (A) IL-10 (*n*=5-6), (B) IL-1β (*n*=5), and (C) IL-6 (*n*=6) assessed by real-time RT-PCR (*n*=10). White columns, sham; black columns, MI; mean±SEM. *P<0.05 versus corresponding sham.

**ONLINE RESOURCE 5**

**
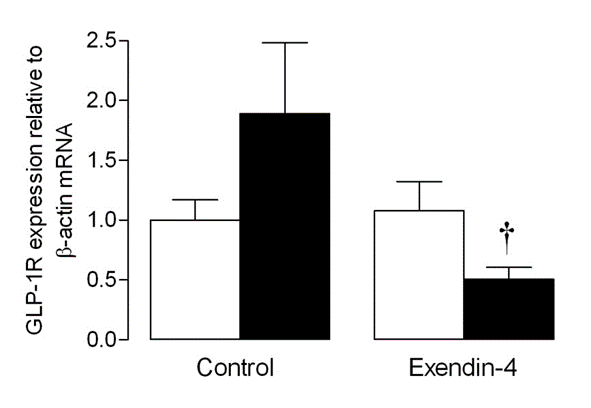
**

Effect of exendin-4 on GLP-1R expression after MI. Graph shows mRNA expression assessed by real-time RT-PCR (*n*=10). White columns, sham, black columns, MI, mean±SEM. ^†^P<0.05, MI exendin-4 versus MI control.

**ONLINE RESOURCE 6**


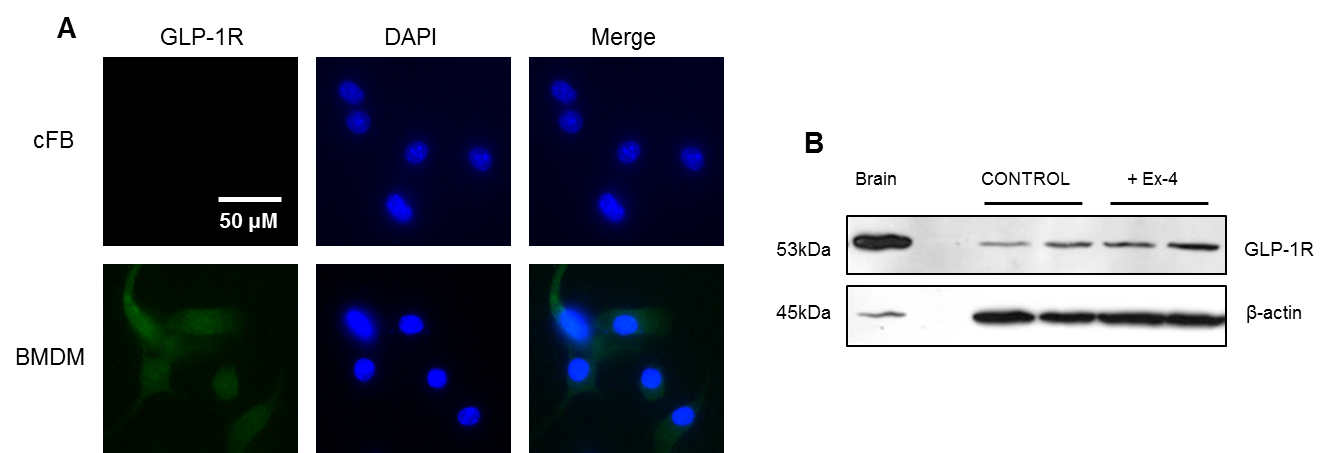


GLP-1R expression in isolated cardiac fibroblasts and BMDM. A: Representative immunocytochemistry images of GLP-1R expression with DAPI nuclear counterstaining in murine cardiac fibroblasts and BMDM. B: GLP-1R protein expression in BMDM (with reference to brain positive control) in the presence/absence of exendin-4 (Ex-4; 10nmol/L) for 24h; blots were stripped and re-probed and normalised to β-actin.
